# Team players and helpers – describing professional identity among finnish physicians in a cross-sectional study

**DOI:** 10.1186/s12909-024-05268-7

**Published:** 2024-03-19

**Authors:** Pyry Mattila, Harri Hyppölä, Teppo Heikkilä, Sami Heistaro, Minna Kaila, Petri Kulmala, Markku Sumanen, Pekka Mäntyselkä

**Affiliations:** 1 Institute of Public Health and Clinical Nutrition, General Practice, University of Eastern Finland, Yliopistonranta 1 C, Kuopio, FI-70211 Finland; 2 Emergency Department, Mikkeli Central Hospital, Mikkeli, Finland; 3 Helsinki University Hospital, Helsinki, Finland; 4 Finnish Medical Association, Helsinki, Finland; 5 Public Health Medicine, University of Helsinki, Helsinki, Finland; 6 Faculty of Medicine and MRC Oulu, University of Oulu, Oulu University Hospital, Oulu, Finland; 7 Faculty of Medicine and Health Technology, Tampere University, Tampere, Finland; 8 Clinical Research and Trials Centre, Wellbeing Services County of North Savo, Kuopio, Finland

**Keywords:** Physicians, Professional identification, Social identity, Professional identity

## Abstract

**Background:**

Every physician has a unique professional identity. However, little is known about the diversity of identities among physicians. This study aimed to quantitatively assess the professional identity of physicians in Finland using descriptions of professional identity.

**Methods:**

This study was part of a larger cross-sectional Finnish Physician 2018 Study. The target population consisted of all Finnish physicians under the age of 70 (*N* = 24,827) in 2018. The sample was drawn from physicians born on even numbered days (*N* = 11,336) using the Finnish Medical Association register. A total of 5,187 (46%) physicians responded. Professional identity was examined by 27 given characterisations using a five-point Likert scale. Multivariate logistic regression was used in assessing how place of work, graduation year and gender were associated with identity descriptions.

**Results:**

The descriptions which most physicians identified with were “member of a working group/team” (82%), “helper” (82%), and “health expert” (79%); the majority reported these as describing them very or quite well. Identity descriptions such as “prescriber of medications” (68% vs. 45%), “prioritiser” (57% vs. 35%) and “someone issuing certificates” (52% vs. 32%) were more popular among junior than senior physicians. The biggest differences between the genders were found in the descriptions “provider of comfort” (62% vs. 40%) and “someone engaged in social work” (45% vs. 25%), with which women identified more frequently than men.

**Conclusions:**

Strong identification as a member of a team is an important finding in the increasingly multiprofessional world of health care. Importantly, most physicians shared several core professional identity descriptions (i.e., helper, health expert) that reflect the traditional image of an exemplary doctor.

**Supplementary Information:**

The online version contains supplementary material available at 10.1186/s12909-024-05268-7.

## Background

A physician possesses different professional roles depending on his or her current position and work history. These changing roles affect a physician’s professional identity. One’s professional identity begins to form from the beginning of medical school – or even in childhood – and keeps evolving throughout one’s career [1]. Over the past few decades, there has been a growing interest in the professional identities of physicians and identities in general [1–3].

The definition of identity varies slightly depending on the branch of science. In the social sciences, identity is a construction of one’s subjective experiences, beliefs, visions, and expectations, which all together form an individual’s identity. This notion has several dimensions such as cultural, sexual, gender, and professional identity. Importantly, identity formation is considered to be a dynamic and constant process rather than a static, unchangeable state [4]. An individual holds multiple identities which are in constant tension with each other [1].

From the broad concept of identity, our paper focuses on the professional identity of physicians, which develops through the process of socialisation. During this process an individual acquires the rules, norms, behaviours, beliefs, and ethics of a profession or an institution [3]. In the course of daily activities, positive and negative feedback constantly develop an individual’s professional identity, and former and current experiences as well as future aspirations have a great effect on identity formation [1]. A mismatch between an individual’s professional identity and a negative or positive experience at work leads to an identity customisation in which this conflict is resolved by customising one’s identity to align with one’s actions [5]. However, sometimes this conflict is not resolved, which can lead to chronic stress and even burnout, neither of which are uncommon among medical professionals [6, 7]. Paradoxically, doctors have a tendency to overlook their own health while treating others. Positive and negative experiences, especially during their early career, might steer a physician away or towards a particular medical specialty [8], substantially influencing the professional identity outcome as well. A physician’s professional identity is hence formed by interacting with patients, colleagues, teachers, and other employers in a multiprofessional environment. In particular, medical teachers and more experienced doctors are thought to play a major role in this process [1, 5].

The literature on physicians’ professional identities has been primarily descriptive and conceptual, and includes calls for acknowledging the importance of professional identity formation during medical school [1, 9–13]. According to our extensive literature search, a lot of qualitative analyses reflecting physician’s professional identity from various perspectives have been reported, especially those focusing on medical students and junior physicians [14–21]. Interestingly, only a few quantitative studies have been published on the professional identity of medical students and even less on specialists or physicians in general [22–27].

In 2010, a Carnegie Foundation Report stated that professional identity formation (PIF) should be a major aim in medical training [28], highlighting the importance and benefits of a strong professional identity during one’s later career. However, these calls seem to include a hidden assumption that there is some kind of general or ideal professional identity that the medical profession shares or at least should share. The Hippocratic Oath [29] and the CANMEDS framework [30] are well-known and widely accepted attempts to capture this identity, in which the former describes the moral and ethical codes for practice and behaviour, and the latter the competencies and key roles of a medical expert: a communicator, a collaborator, a leader, a health advocate, a scholar, and a professional. Nevertheless, despite these descriptions of the ideal professional traits of physicians, there is a lack of nation-wide quantitative studies trying to illuminate the subjective professional identity of the medical profession. Moreover, given that physicians are still one of the most respected professions of a society [31, 32], there is still scant understanding of how physicians describe themselves as health professionals. Consequently, using a quantitative methodology and a large Finnish Physician questionnaire study [33], this research examines the professional identities of physicians.

In the present study, our aims were [1] to explore whether there is any widely shared professional identity among physicians [2], to assess which descriptions of a professional identity best matched Finnish physicians, and [3] how these descriptions were associated with the background of a given physician.

## Methods

This study was a part of the *Finnish Physician 2018* -survey [33]. The survey is the latest from a chronological set of questionnaire studies, the first of which originated in the 1980s (the Young Physician − 88 study [34]). Since then, similar cross-sectional surveys have been conducted every five years. Along with the background characteristics of the respondents, the themes cover e.g. current work status, work satisfaction, opinions on past basic medical training, specialist training, recertification, values and identity [35]. The recent questionnaire included 55 questions overall.

In this paper, we focus on the results regarding the professional identity of physicians and report the results according to the STROBE guidelines (Supplementary material). The question concerning the professional identity was developed by a group of medical professors and other experts. This was regarded as the best solution at the time. The identity descriptions were selected with the aim of capturing the diverse nature of medical profession identity roles from different angles, using the – at that time – current literature published on the professional identities of physicians. Since then, the questions have remained almost the same for three decades.

**Fig. 1:**
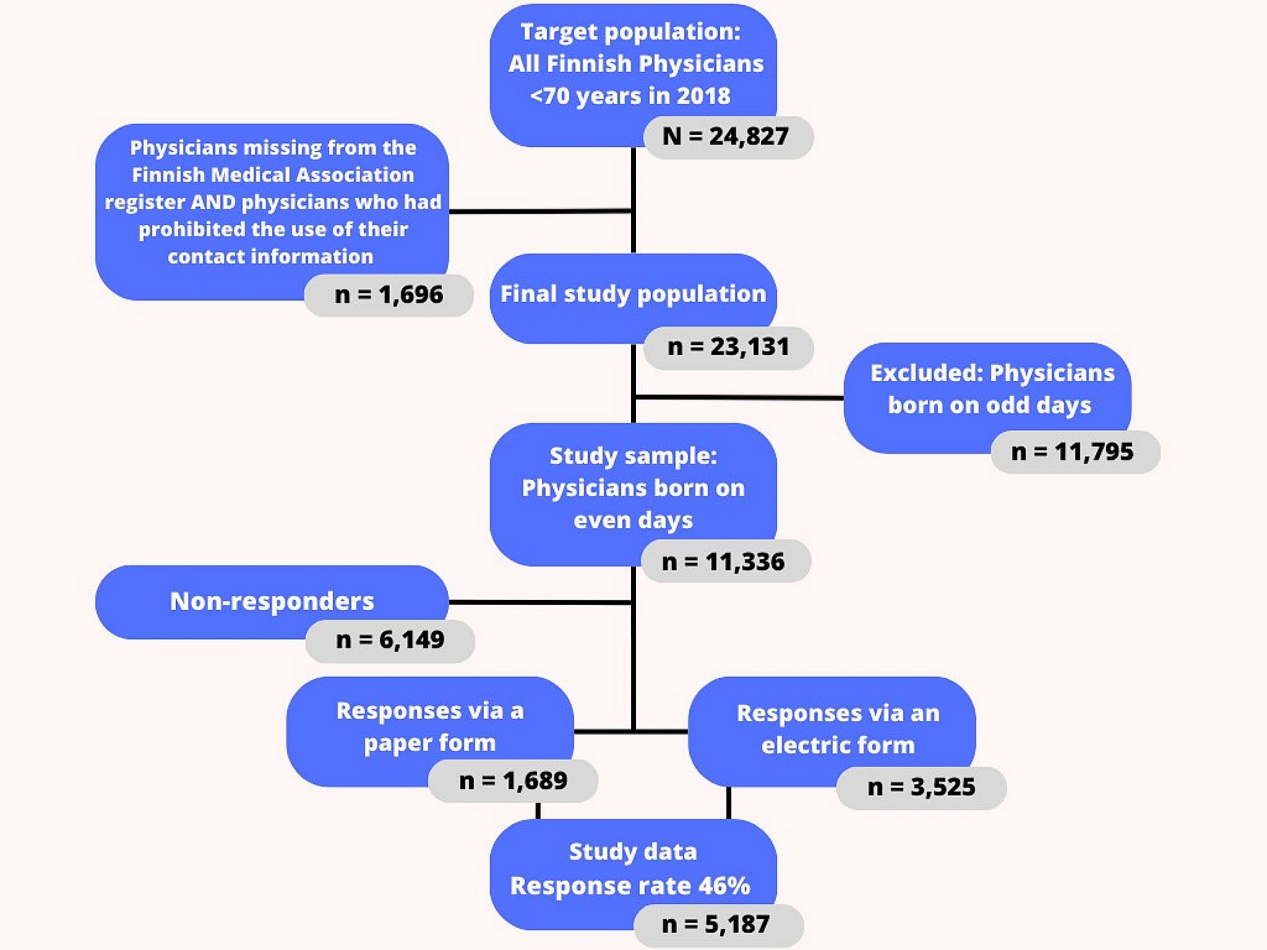
Flowchart of the finnish physician 2018 study population. the information was collected from the registries of the finnish institute for health and welfare (THL) and the finnish medical association (FMA)

Our study population comprised all licenced Finnish physicians under the age of 70 (*n* = 24,827) in 2018 (Fig. 1). The sample was drawn from the Finnish Medical Association’s member register, which includes information on most of the physicians in Finland as well as their contact details. Individuals who had prohibited the use of their information were excluded. Thereafter, the target population was comprised of 23,131 (93%) Finnish physicians.

Physicians who were born on even numbered days (*n* = 11,336) were chosen for the study sample. The data were gathered between September 4th and December 7th, 2018. A total of 5,214 physicians answered the questionnaire: 3,525 using an online form and 1,689 a paper form. If a respondent had answered both (*n* = 27), the online form was accepted. Overall, the response rate was 46% (*n* = 5,187). The questionnaire was written and answered in Finnish. After the data collection, the questionnaire was translated into English by an authorised translator to ensure correspondence (Questionnaire, Appendix).

**Fig. 2:**
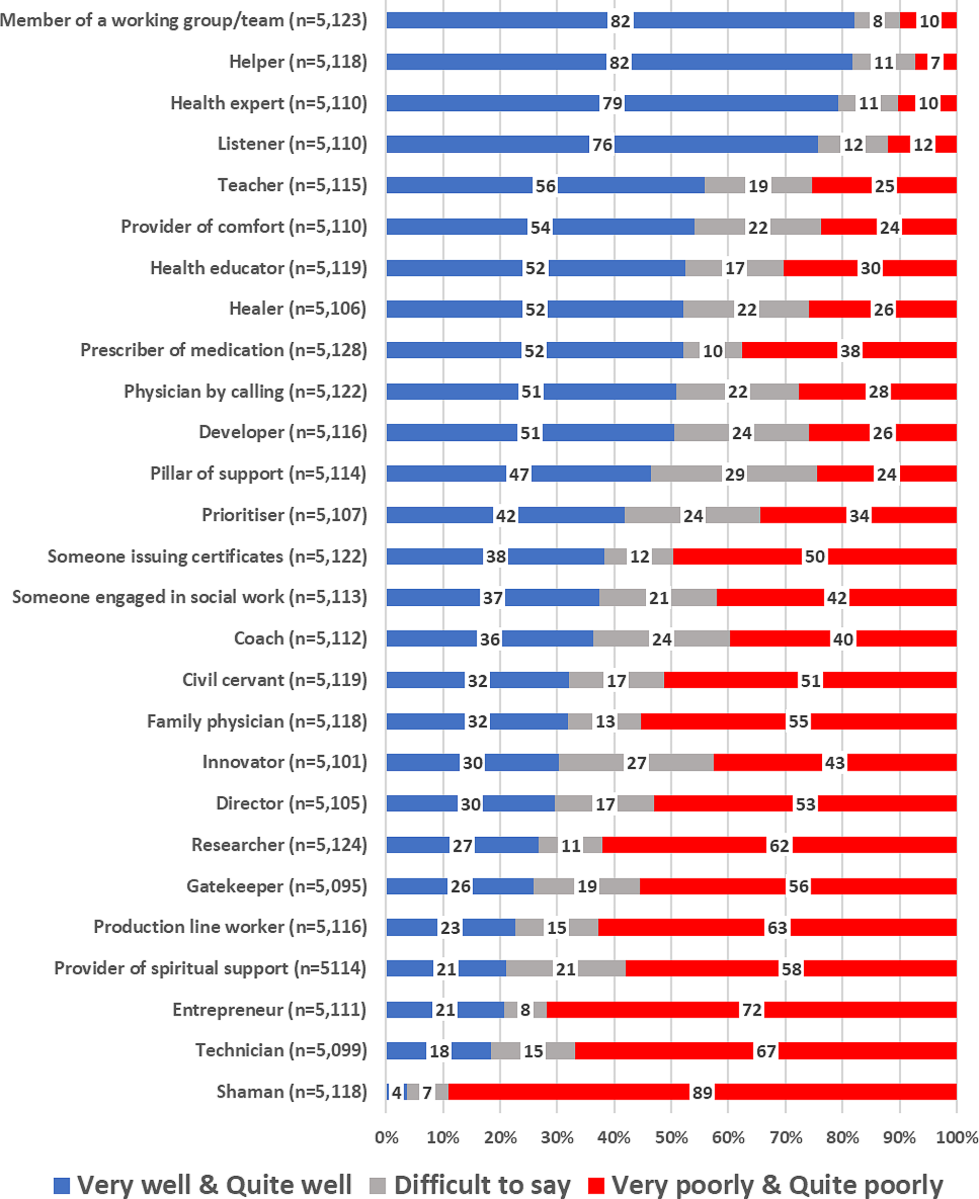
“How well do the following descriptions of the work of a physician match you as a physician?”. Proportions of Finnish physicians (*n* = 5,187) according to descriptions of professional identity

The background variables used in this paper included gender (*woman, man*), age, year of graduation, place of work (*hospital, primary health care)*, specialisation status (*non-specialist, specialist*). The professional identity of physicians was inquired with the following question: “How well do the following descriptions of the work of a physician match you as a physician?”. The question included 27 descriptions (Fig. 2) and the respondents had to answer the best fit option for all of them, using a Likert five-point scale: “*very well*”, “*quite well*”, “*difficult to say*”, “*quite poorly*”, and “*very poorly*”. These options were combined into three separate categories: “*very well or quite well*”, “*difficult to say”* and *“quite poorly or very poorly*”. To answer the question “How well do the following descriptions…”, we focused only on the participants who had answered “*very well or quite well*” in our reporting. Thus, these three categories were further combined into a binary category: “*very well or quite well*” and “*difficult to say or quite poorly or very poorly”*.

### Statistics

Frequencies and proportions of respondent characteristics were calculated. Median and interquartile range (IQR) were calculated for age, the only continuous variable, as the data were not normally distributed. The respondents were grouped by gender (*woman, man*), place of work (*primary health care centre, hospital*), and graduation year (*junior physicians, senior physicians*). A respondent was classified as a *junior physician* if he or she had graduated between 2007 and 2018 whereas *senior physicians* had graduated before 2007. A chi-squared test was used to test the statistical significance, and *p* < 0.05 was considered significant.

Each description of professional identity was cross tabulated separately by place of work, graduation year and gender. For each of the descriptions, the proportions (%) of respondents who answered “very well” or “quite well” to the question “How well do the following descriptions of the work of a physician match you as a physician?” were calculated. In a corresponding manner, a multivariate binary logistic regression analysis, adjusted for place of work, graduation year and gender, was conducted for each professional identity description. Missing data were excluded pairwise in the analysis. The results from each multivariate analysis are presented separately (Tables 1, 2 and 3) for each covariate (place of work, graduation year and gender) using odds ratios (OR) with 95% confidence intervals (95% CI). The analysis was executed using IBM’s SPSS Statistics version 27.

**Table 1:** Professional identity descriptions among physicians in hospital and primary health care settings. The proportions of physicians who answered “very well” or “quite well” to the question “How well do the following descriptions of the work of a physician match you as a physician?”, and the corresponding associations (odds ratios, primary health care as a reference group)

Description	Hospital	Health care centre	Odds Ratio (95% CI)*	*p*-value*
	*(n = 2,148)*	*(n = 992)*		
	%	%		
Member of a working group/team	90.0	84.2	1.85 (1.47–2.34)	**< 0.001**
Helper	80.0	87.8	0.61 (0.49–0.76)	**< 0.001**
Health expert	73.0	88.5	0.37 (0.30–0.46)	**< 0.001**
Listener	66.3	89.6	0.24 (0.19–0.30)	**< 0.001**
Teacher	60.0	54.1	1.20 (1.02–1.40)	**0.025**
Developer	54.6	44.8	1.36 (1.16–1.59)	**< 0.001**
Healer	49.6	50.9	0.87 (0.74–1.02)	0.079
Physician by calling	48.6	53.2	0.84 (0.72–0.99)	**0.034**
Prescriber of medication	46.3	72.6	0.36 (0.31–0.43)	**< 0.001**
Provider of comfort	46.0	70.5	0.39 (0.33–0.46)	**< 0.001**
Prioritiser	45.3	52.1	0.86 (0.74–1.01)	0.063
Pillar of support	40.3	54.4	0.56 (0.47–0.65)	**< 0.001**
Health educator	40.0	69.3	0.31 (0.27–0.37)	**< 0.001**
Civil servant	35.5	41.8	0.73 (0.63–0.86)	**< 0.001**
Coach	34.0	35.0	0.84 (0.71–0.99)	**0.034**
Researcher	33.3	12.4	3.41 (2.75–4.22)	**< 0.001**
Someone engaged in social work	32.0	57.0	0.40 (0.34–0.47)	**< 0.001**
Director	31.5	25.2	1.19 (1.00-1.42)	0.053
Innovator	29.9	22.6	1.28 (1.06–1.53)	**0.009**
Someone issuing certificates	27.3	64.2	0.23 (0.20–0.27)	**< 0.001**
Production line worker	26.3	29.5	0.91 (0.76–1.08)	0.273
Technician	25.7	11.3	2.70 (2.15–3.39)	**< 0.001**
Gatekeeper	24.5	45.3	0.43 (0.36–0.50)	**< 0.001**
Family physician	17.3	62.9	0.12 (0.10–0.15)	**< 0.001**
Provider of spiritual support	15.8	28.5	0.46 (0.38–0.56)	**< 0.001**
Entrepreneur	11.2	6.0	1.63 (1.20–2.20)	**0.002**
Shaman	3.5	4.5	0.74 (0.50–1.09)	0.132

*Binary logistic regression analysis (refence group health care centre) adjusted for gender and graduation year

**Table 2:** Professional identity descriptions among senior (graduated before 2007) and junior (graduated between 2007–2018) physicians. The proportions of physicians who answered “very or quite well” to the question “How well do the following descriptions of the work of a physician match you as a physician?”, and the corresponding associations (odds ratios, junior physicians as a reference group)

Description	Senior physicians	Junior physicians	Odds Ratio (95% CI)*	*p*-value*
	*(n = 3,645)*	*(n = 1,525)*		
	%	%		
Member of a working group/team	80.1	86.9	0.90 (0.72–1.14)	0.387
Helper	80.1	85.8	0.78 (0.64–0.95)	**0.015**
Health expert	77.6	83.1	0.72 (0.60–0.87)	**0.001**
Listener	75.3	76.6	1.07 (0.90–1.28)	0.433
Teacher	57.8	51.2	1.46 (1.26–1.70)	**< 0.001**
Healer	55.4	44.3	1.50 (1.30–1.74)	**< 0.001**
Developer	54.0	42.3	2.00 (1.72–2.32)	**< 0.001**
Physician by calling	53.1	45.4	1.29 (1.11–1.50)	**0.001**
Provider of comfort	51.7	59.7	0.81 (0.69–0.94)	**0.006**
Health educator	51.4	55.0	0.91 (0.78–1.06)	0.230
Pillar of support	48.5	41.8	1.44 (1.24–1.68)	**< 0.001**
Prescriber of medication	45.1	68.4	0.46 (0.39–0.54)	**< 0.001**
Coach	40.7	25.8	2.11 (1.80–2.49)	**< 0.001**
Prioritiser	35.3	57.3	0.49 (0.42–0.57)	**< 0.001**
Innovator	34.5	20.2	2.26 (1.89–2.70)	**< 0.001**
Director	32.6	22.6	1.79 (1.51–2.13)	**< 0.001**
Someone issuing certificates	32.4	52.4	0.50 (0.42–0.58)	**< 0.001**
Someone engaged in social work	31.8	50.9	0.54 (0.46–0.63)	**< 0.001**
Family physician	31.5	33.1	1.18 (0.98–1.41)	0.075
Civil servant	30.0	36.9	1.05 (0.90–1.23)	0.520
Researcher	26.9	26.0	1.03 (0.87–1.22)	0.747
Entrepreneur	25.5	8.8	2.63 (1.95–3.55)	**< 0.001**
Provider of spiritual support	22.0	18.8	1.27 (1.05–1.54)	**0.013**
Gatekeeper	21.1	37.4	0.62 (0.53–0.72)	**< 0.001**
Production line worker	17.9	33.5	0.57 (0.48–0.67)	**< 0.001**
Technician	17.0	21.6	0.70 (0.58–0.84)	**< 0.001**
Shaman	3.4	4.1	0.78 (0.53–1.14)	0.193

*Binary logistic regression analysis (refence group junior physicians) adjusted for gender and place of work

**Table 3:** Professional identity descriptions of physicians according to gender. The proportions of physicians who answered “very well” or “quite well” to the question “How well do the following descriptions of the work of a physician match you as a physician?”, and the corresponding associations (odds ratios, women as a reference group)

Description	Men	Women	Odds Ratio (95% CI)*	*p*-value*
	*(n = 1,824)*	*(n = 3,270)*		
	%	%		
Member of a working group/team	78.0	84.6	0.60 (0.47–0.75)	**< 0.001**
Helper	76.4	85.1	0.64 (0.53–0.78)	**< 0.001**
Health expert	75.2	81.4	0.75 (0.62–0.90)	**0.002**
Listener	65.8	81.3	0.47 (0.40–0.56)	**< 0.001**
Teacher	55.8	56.0	1.01 (0.87–1.18)	0.876
Healer	53.5	51.3	1.28 (1.10–1.49)	**0.002**
Developer	51.6	50.0	1.00 (0.85–1.16)	0.967
Physician by calling	43.7	55.1	0.58 (0.50–0.68)	**< 0.001**
Prescriber of medication	42.7	57.3	0.62 (0.53–0.73)	**< 0.001**
Health educator	41.1	58.8	0.48 (0.40–0.56)	**< 0.001**
Provider of comfort	39.8	62.4	0.45 (0.39–0.53)	**< 0.001**
Prioritiser	39.1	43.4	0.93 (0.79–1.08)	0.334
Pillar of support	38.0	51.4	0.67 (0.57–0.79)	**< 0.001**
Coach	37.6	35.5	1.22 (1.04–1.43)	**0.016**
Innovator	35.4	27.4	1.32 (1.11–1.57)	**0.001**
Director	35.4	26.6	1.39 (1.18–1.64)	**< 0.001**
Researcher	31.6	23.8	1.32 (1.11–1.57)	**0.002**
Civil servant	31.0	32.7	1.09 (0.93–1.28)	0.274
Entrepreneur	28.4	16.3	1.95 (1.52–2.50)	**< 0.001**
Someone issuing certificates	27.5	44.3	0.49 (0.41–0.58)	**< 0.001**
Family physician	26.3	35.3	0.56 (0.47–0.68)	**< 0.001**
Technician	26.1	14.0	2.02 (1.69–2.42)	**< 0.001**
Someone engaged in social work	24.5	44.8	0.42 (0.35–0.50)	**< 0.001**
Gatekeeper	23.3	27.5	0.94 (0.79–1.11)	0.447
Production line worker	22.9	22.4	1.09 (0.92–1.29)	0.320
Provider of spiritual support	16.0	23.8	0.70 (0.57–0.86)	**< 0.001**
Shaman	4.5	3.1	1.78 (1.22–2.60)	**0.003**

*Binary logistic regression analysis (refence group women) adjusted for graduation year and place of work

## Results

### Characteristics of the study population

Of the total of 5,187 respondents, there were 3,270 (64%) women and 1,824 men (36%), and the median (IQR) age was 49 years (37.0, 60.0) (Table 4). One fifth (19%) of the physicians were under the age of 35 years and 12% were at least 65 years. The distribution of ages in the other ten-year intervals was quite similar. The largest group was physicians between the ages of 55 and 64 years, comprising 26% of the sample. Two thirds of the physicians (68%) were specialists. When compared to the study sample, women responded to the questionnaire more frequently (49%) than men (34%) (Table 4). The highest response rate was among the oldest respondents (> 65 years) and lowest between 35–44-year-olds. Specialists answered more often (48%) than non-specialist physicians (41%). Beyond the characteristics of the participants and study sample, we have not further examined the reasons for non-participation.

**Table 4:** Characteristics of the study sample and respondents by gender, age, year of graduation, place of work and specialisation

	Study sample *n* (%)	Respondents *n* (%)	Response rate %
	**11,336 (100)**	**5,187 (100)**	**46**
Gender			
Women	6,681 [59]	3,270 (64)	**49**
Men	4,655 [41]	1,824 [36]	**39**
Age			
<35-years old	2,275 [20]	989 [19]	**43**
35–44-years old	2,689 [24]	1,077 [21]	**40**
45–54-years old	2,470 [22]	1,095 [21]	**44**
55–64-years old	2,763 [24]	1,351 [26]	**49**
65–69-years old	1,139 [10]	628 [12]	**55**
Year of graduation			
Juniors (graduated between 2007–2018)	N/A	1,525 [29]	**N/A**
Seniors (graduated before 2007)	N/A	3,645 (71)	**N/A**
Place of work			
Hospital	N/A	2,148 (68)	**N/A**
Primary health care	N/A	992 [32]	**N/A**
Specialisation			
Yes	7,229 (64)	3,484 (68)	**48**
No	4,107 [36]	1,674 [32]	**41**

*Missing data: gender n = 93, age n = 47, year of graduation n = 17, place of work = 498, specialisation n = 29.*

### Professional identities

The descriptions which most physicians identified with were “*member of a working group/team*”, “*helper*”, and “*health expert”*, with the majority of the respondents saying that these described them very well or quite well: 82%, 82% and 79%, respectively (Fig. 2). The descriptions of “*shaman*” (4%), “*technician*” (18%) and “*entrepreneur”* (21%) were least popular among the respondents.

Physicians working at hospitals tended to identify themselves less often as “*family physician*”, “*someone issuing certificates*” and “*health educator*” when compared with the physicians working in primary health care: 17% vs. 63%; 27% vs. 64%; 40% vs. 69% respectively (Table 1). Similarly, the three lowest odds ratios for a description matching a physician working at a hospital were “*family physician*” OR 0.12 (95% CI 0.10–0.15, *p* < 0.001), “*someone issuing certificates*” OR 0.23 (95% CI 0.20–0.27, *p* < 0.001) and “*listener*” OR 0.24 (95% CI 0.19–0.30, *p* < 0.001), when compared to primary health care centre physicians. Up to 33% of physicians who worked at hospitals regarded themselves as “*researchers*”, in comparison to 12% of physicians who worked in primary health care centres. Likewise, the highest odds ratios were for the descriptions: “*technician*” (OR 2.70, 95% CI 2.15–3.39, *p* < 0.001) and “*researcher*” (OR 3.41, 95% CI 2.75–4.22, *p* < 0.001).

The study showed that professional identity descriptions such as “*prescriber of medications*”, “*prioritiser*” and “*someone issuing certificates*” occurred more often among junior physicians. Two thirds of junior responders reported that the descriptions “*prescriber*” represented them very well or quite well, while 45% of senior physicians identified themselves similarly. Descriptions such as “*entrepreneur*”, “*coach*” and “*innovator*” were more frequent among the senior physicians’ group. The odds ratios showed a similar pattern, when comparing junior and senior physicians (Table 2).

The biggest differences between the genders were found in the descriptions “*provider of comfort*”, “*someone engaged in social work*” and “*health educator*”, with which women identified more frequently than men (Table 3). The study found that 62% of women identified themselves as “*provider of comfort*”, compared with 40% among male respondents. The three lowest odds ratios for a description matching a female physician were “someone engaged in social work” OR 0.42 (95% CI 0.35–0.50, *p* < 0.001), “provider of comfort” OR 0.45 (95% CI 0.39–0.53, *p* < 0.001) and “health educator” OR 0.48 (95% CI 0.40–0.56, *p* < 0.001), when compared to men. Descriptions such as “*entrepreneur”*, “*technician*” and “*director*” were more common among male respondents. Similarly, the highest odds ratios were for the descriptions: “technician” (OR 2.02, 95% CI 1.69–2.42, *p* < 0.001) and “researcher” (OR 2.02, 95% CI 1.69–2.42, *p* < 0.001).

## Discussion

Finnish physicians identified themselves primarily as team members, helpers, and health experts, reflecting the traditional image of the ideal descriptions of a physician. Despite the medical profession’s gradual shift towards narrower specialties and subspecialties, there are still shared ‘core’ identity descriptions that almost every physician agrees with, which is the foremost finding of the study. The respondents were surprisingly homogenous with regard to these descriptions, and the result was consistent throughout the comparisons between gender, experience, and place of work. As professional identity formation has been highlighted as an important aim in medical education [28, 36], these shared core identity descriptions align closely with the ideal identities and could be recognised as a common foundation for physicians, and be further emphasised in basic and specialist training – the critical phases of professional identity formation. Nevertheless, despite the well-known definitions of an ideal physician, there is not only one ideal professional identity of a physician. In particular, two ‘ideal’ doctors could simultaneously possess different professional identities and still be ideal from the perspective of a patient, the medical profession and/or society. The virtues of an excellent physician might be the same, but the professional identity outcomes still vary between individuals.

In this regard, Frost and Regehr have argued that medical students face two separate and somewhat opposing discourses, which can cause a conflict in their identity formation during basic training [2]. Nowadays, medical schools tend to emphasise the importance of *diversity* among medical students: the benefit of having individuals from various backgrounds who match the heterogeneous patient population and hence better understand their needs. However, medical education forms another conflicting discourse, *standardisation*, in which students are encouraged to behave and act in a particular manner, respecting the traditions of the medical profession, in addition to mastering sufficient skills and knowledge. To some extent, these two discourses, *diversity* and *standardisation*, are constantly in tension in the formation of professional identity. This might be especially challenging for individuals who do not represent the stereotypical image of a doctor. To a certain degree, our results illustrated the analogous variation: a pattern of similarity but also diversity in terms of professional identity descriptions.

In the Young physician − 88 survey, physicians under 40 years old working in primary care identified themselves more often with the descriptions “*production line worker*”, “*certificate writer*”, “*prescriber*” and “*social worker*”, when compared to physicians working in hospitals, who in turn were more “*healers*” and “*technicians*” [22]. After 30 years and concerning all Finnish physicians, our results were in concordance with this former finding. According to our analysis, the biggest differences in professional identities were found between hospital and primary health care, even when the analysis was adjusted for gender and graduation year. On one hand, it seems that one’s place of work still has a greater influence on a physician’s identity than gender or work experience. This result might indicate that one of the most important factors affecting professional identity comes from the specialty-specific type of interaction between doctor and patient. Given that general practitioners (GP) and anaesthesiologists have distinct roles in a health care system, it is probable that these roles have an effect on their professional identity as well [4]. Likewise, medical specialties have their own socialisation in becoming a full member of that particular department with its own behaviours and ideals of a good doctor, most likely influencing professional identity [4, 37]. On the other hand, while personality traits have been recognised as important factors when choosing a medical speciality, there is a high chance that physicians with distinct personalities – and also professional identities – choose different medical specialities early in their career [38]. Young generations of physicians seem to emphasise the importance of a good work-home balance, whereas men and women tend to have different expectations on what this work-home balance means. Therefore, both age and gender impact one’s choice of specialty [39]. In conclusion, the effect of working sector might be smaller than our results implicate, and the causal pathway a mixture of many factors.

When compared to seniors, junior physicians reported being more clearly ‘*prescribers of medication’*, ‘*prioritisers’*, and ‘*someone issuing certificates’*. At first glance, the differences might illustrate the generational gap between younger and older physicians. However, another explanation is that senior physicians tend to work more often in management roles that do not include as much clinical practice, especially when compared to the early years of a medical career. Also, when comparing doctors with unequal amount of experience, these differences might be the result of a completely natural growing process as a physician.

Interestingly, these three descriptions that exhibited large differences (i.e., ‘*prescribers of medication’*, ‘*prioritisers’*, ‘*someone issuing certificates’)* also had a somewhat negative undertone, perhaps indicating excessive workload or a sense of urgency leading to cynical attitudes towards their daily practice. To support this argument, there are two factors that in combination might explain this finding. First, qualitative studies in Europe have suggested that, among other things, clinical practice in primary care has changed increasingly towards writing of certificates and other bureaucracy, leading to a higher workload and greater dissatisfaction [40–42]. Second, younger physicians typically begin their career in primary care in Finland. Thus, these two notions could explain the differences in professional identity in terms of graduation year. In addition, one of many key roles of a GPis to act as a gatekeeper trying to promote the clever use of common resources and to avoid an overdiagnosis [43]. Gatekeeping involves the ability to prioritise in many ways, which requires experience, a strong medical background and good communication skills. However, facing these situations without sufficient support from colleagues and public might cause negative feelings and distress, which is especially relevant when it comes to young doctors. Nevertheless, when looking from another perspective, the ability to prioritise is also a vital skill for a successful career, and our finding might be a mere reflection of an improved understanding of that fact among younger physicians.

With regard to the gap between juniors and seniors, every generation of physicians develops its own way of practicing and becoming members of the profession [44]. As mentioned earlier, the increasing value placed on leisure time and a wish to succeed in other facets of life is becoming more widespread, especially among younger physicians [39, 45, 46]. According to some views, this has reduced the necessity of forming strong physician identities that persist outside of working hours [2, 45]. In addition, the inheritability of the physician’s discipline has increased over the past two generations in Finland, which is a similar trend in other Nordic countries [47–49]. Therefore, if students tend to choose the same profession as their parents, it is evident that parents are also influencing the professional identity of the next generation in many ways: as role models, mentors, and/or teachers. This adds another important factor to the process of professional identity formation that should be acknowledged.

The respondents seemed to identify themselves more regularly as a “*member of a working group/team*”, rather than a “*director*”. This is a particularly important finding as a physician’s work is largely interprofessional collaboration – and probably will be even more in the future, following a rather positive change [50–52]. Furthermore, the respondents may have understood the term “director” in different ways, e.g., some of them as a formal position in the organisation and some as a medically responsible team member. However, despite a strong feeling of being an equal member of a team, legal responsibility is still not shared equally: physicians still ultimately bear the legal consequences. Thus, despite the valid arguments for collective responsibility in health care [53], the popular identification of being ‘just’ part of a team should not lead to a false feeling of shared responsibility.

With regard to communication with the team or a patient, the ability to listen is an essential trait [54]. According to the Clinician of the Future survey (2022), listening is still an increasingly important part of physicians’ clinical work even though technological development has changed the possibilities for interaction [55]. Similarly, our analysis showed that the majority of physicians reported the description of a listener describing themselves well, which is important given that one of the reasons for patient dissatisfaction is the physician’s lack of listening during an appointment [56]. If we look at our results the other way round, surprisingly many (24%) did not subjectively identify themselves as listeners. Despite the stereotypical misbehaving physicians in popular culture (e.g., Dr. House, Doc Martin), the ability to listen should be a standard skill rather than the skill of a good doctor. Interestingly, Gude et al. (2017) have showed that there is a clear discrepancy between the self-assessed scores and trained observers scores when it comes to communication skills [57]. Therefore, the subjective professional identification might not be objectively valid.

Finally, women identified themselves more frequently as a “*provider of comfort*”, a “*someone engaged in social work*” and a “*health educator”* compared to men. As noted above, this result could be partially explained by the difference in work environments, and it also reflects the difference in the patient-physician relationship. Male physicians more often tend to work at hospitals as operating specialists and in internal medicine when compared to females, who work more in primary care as GPs in Finland [58]. However, the finding was similar when the analysis was adjusted for graduation year and place of work. Nevertheless, these characterisations describe a slightly softer and caring side of physician work that has traditionally been associated more with women than with men. Of the descriptions above, health education in particular is still an important part of a physician’s profession – regardless of specialty or department. In addition to the usual situation between a physician and a patient, health education also occurs in variety of settings, and it involves interactions with colleagues, other health personnels and the public.

### Strengths and limitations

The foremost strength of our study was that we were able to examine the professional identity of Finnish physicians with a large set of quantitative data. To our knowledge, there have not been any attempts to capture the professional identity of physicians at this magnitude, and we believe that our data adequately represent the target population. The response rate of our study was 46%, which is higher than that of many comparable surveys of this size (see refs [59–61]). However, this still presents a potential limitation for the present study. Also, it is probable that non-respondents might characterise different patterns of professional identity which this paper was not able to capture. Open questions with a qualitative study design could provide a better understanding of the variety of issues related to identity descriptions. Using the 27 predefined descriptions necessarily narrows the scope, and every respondent understood these descriptions from their own perspective. Nevertheless, despite these limitations, we believe that our results are valid and reflect the professional identity of Finnish physicians.

The study questionnaire was originally created some three decades ago by a group of experienced researchers for practical purposes, with the aim to better understand how physicians work and who they are, without a specific theoretical background on identity descriptions [34]. The survey has been repeated over the years without much variability in the questions. It seems reasonable that sufficient validation has been gained. This also applies to the question concerning professional identity, the responses to which are reported here.

## Conclusions

A great majority of the physician respondents of the present study shared several core identity descriptions that reflect the traditional image of an exemplary doctor [30]. Regardless of the diverse backgrounds of physicians, the descriptions as a *member of a group* and a *helper* connected the majority of the respondents to each other, which was a major finding in terms of the feeling of collegiality, professionalism, and unity. If we want to restore professional identity formation as an important aim in medical education [62], we argue that instead of focusing on the differences between generations or specialities, these strongly shared identities should be given a larger role in the current medical discourse, education and training. Simultaneously, there is clearly value in the differences and diversity of physicians, reflecting that of the patients, who are similarly diverse in backgrounds, personalities, and needs.

### Electronic supplementary material

Below is the link to the electronic supplementary material.

Supplementary Material 1

Supplementary Material 2

## Data Availability

The datasets used and/or analysed during the current study are available from the corresponding author on reasonable request.

## Abbreviations

PIF: Professional identity formation
