# Supplementary material for: Team players and helpers – describing professional identity among finnish physicians in a cross-sectional study
Source: BMC Med Educ. 2024 Mar 19;24:304. doi: 10.1186/s12909-024-05268-7 (PMC10949613; doi:10.1186/s12909-024-05268-7)
Supplement: Supplementary file 1 — Supplementary Material 1 [file 12909_2024_5268_MOESM1_ESM.docx]

PHYSICIAN 2018

---------------------------------------------------------------------------------------------------------------------

ANSWERING INSTRUCTIONS:

Check the option that best reflects your opinion or write the information requested in the space given. Use whole numbers for numerical answers and leave empty areas blank (no dashes). Only choose one option unless otherwise instructed.

**Graduating as a physician**

1. In which year did you graduate as a physician (Licentiate of Medicine)? ___________________

2. In which year did you start studying medicine? _________________

3. From which University did you graduate as a physician?

- University of Helsinki
- University of Kuopio / University of Eastern Finland
- University of Oulu
- University of Tampere
- University of Turku
- Abroad, please specify the country and university ____________

**Choosing to study medicine**

4. To what extent did the following factors influence your decision to start studying medicine?

1 = Not at all, 2 = A little, 3 = Somewhat, 4 = Quite a lot, 5 = Very much

- Vocation/calling
- Respected profession
- Good pay
- Having a physician in your family or among close relatives
- Interest in people
- Shortage of physicians
- Achievements at school
- Your or your close relative’s illness
- Careers guidance
- Broad range of different opportunities
- Interest in research

5. If you were starting your studies now, would you choose to be a physician?

- No
- Yes

**Your work situation**

6. What is your work situation at the moment?

- Working
- Full-time pensioner, working
- Full-time pensioner, not working
- On maternity, paternity or child-care leave, working
- On maternity, paternity or child-care leave, not working
- Not working for other reasons

If you are not currently working, jump to question 14.

7. What is your main place of work?

- University hospital
- Other hospital run by a municipality / joint municipal board
- Health centre – employed by a municipality
- Health centre – employed by other than a municipality
- Municipal occupational health care
- Private-sector occupational health care
- Private medical clinic / medical centre / hospital, private practice
- University
- Government office or public body
- Foundation, association or organisation
- Pharmaceutical industry
- Employment company / agency (‘outsourced’ physicians not working in health centres)
- Some other place, please specify _______

8. What is your job title in your main occupation? Select the nearest option.

- Medical Director, Director, Chief Physician
- Head of Department, Deputy Chief Physician
- Specialist, Senior Ward Physician
- Specialist trainee, specialist trainee in General Practice
- Health Centre Physician
- Occupational Health Physician, Occupational Health Physician in charge
- Medical adviser
- Private medical practitioner
- Professor, Associate Professor
- Clinical Instructor, University Lecturer, University Instructor
- Researcher
- Some other title, please specify ______

9. Which of the following are you at the moment?

- Permanently employed
- Employed on a fixed-term contract
- Private practitioner
- Self-employed

10. Is your main occupation

- Full-time
- Part-time, working __________ hours per week

11. Number of inhabitants in the municipality where your main practice is based?

- Less than 10,000
- 10,000 to 19,999
- 20,000 to 49,999
- 50,000 to 99,999
- 100,000 to 499,999
- 500,000 or more

12. In which hospital district is your current place of work located?

- Åland
- South Karelia
- South Ostrobothnia
- South Savo
- Hospital District of Helsinki and Uusimaa (Helsinki)
- Hospital District of Helsinki and Uusimaa (Uusimaa)
- East Savo
- Kainuu
- Kanta-Häme
- Central Ostrobothnia
- Central Finland
- Kymenlaakso
- Lapland
- Länsi-Pohja
- Pirkanmaa
- North Karelia
- North Ostrobothnia
- North Savo
- Päijät-Häme
- Satakunta
- Vaasa
- Varsinais-Suomi
- Abroad

**Physician’s work and profession**

13. How satisfied are you with your **current work** on the whole?

- Very dissatisfied
- Fairly dissatisfied
- Hard to say
- Fairly satisfied
- Very satisfied

14. How satisfied are you with **your choice of profession**?

- Very dissatisfied
- Fairly dissatisfied
- Hard to say
- Fairly satisfied
- Very satisfied

15. How well do the following descriptions of the work of a physician match you as a physician?

1 = Very poorly, 2 = Rather poorly, 3 = Hard to say, 4 = Rather well, 5 = Very well

- Healer
- Technician
- Shaman
- Teacher
- Family physician
- Health educator
- Researcher
- Civil servant
- Entrepreneur
- Director
- Health expert
- Someone issuing certificates
- Prescriber of medication
- Production line worker
- Physician by calling
- Helper
- Provider of comfort
- Pillar of support
- Listener
- Someone engaged in social work
- Provider of spiritual support
- Gatekeeper
- Member of a working group/team
- Prioritiser
- Developer
- Coach
- Innovator

16. What kind of work as a physician would you most like to do?

- Primary care physician
- Hospital physician
- Occupational health physician
- Private medical practitioner
- Research
- Teaching
- Administration and management
- Other expert work
- Other, please specify __________
- Don’t know

If you graduated as a physician before 2007, please jump to question 23.

**Basic medical education**

17. How much instruction and guidance on the following topics did you receive during your basic medical education?

1 = Far too little, 2 = Too little, 3 = The right amount, 4 = Too much, 5 = Far too much

Medical knowledge

- Diagnosis and treatment of diseases
- Examination and treatment procedures
- Patient safety

Learning new things

- Information search skills
- Critical evaluation of information
- Research
- Teaching

Interaction skills

- Meeting the patient
- Patient-centredness (patient’s needs and overall situation)
- Interacting with the patient’s relatives
- Written communication (patient records)

Collaboration skills

- Working in a group
- Group leadership
- Presentation skills
- Consultation skills
- Multidisciplinary collaboration

Professional attitude

- The profession’s values and commitment to those values
- Recognising the limits of my skills and acting accordingly
- Ethical considerations

Executive skills

- Effective use of time and prioritising different activities
- Health economics (appropriate use of resources)
- Personal professional advancement

Health promotion

- Health guidance and supporting patients in self-management
- Patient guidance methods
- Health promotion among the population

18. How much instruction and guidance on the following topics did you receive during your basic medical education?

1 = Far too little, 2 = Too little, 3 = The right amount, 4 = Too much, 5 = Far too much

- Collaboration between different specialties
- Collaboration between primary care and specialist care
- Collaboration with social services
- Collaboration with different service sectors in the municipality (other than social services)
- Multiculturalism
- Physicians’ electronic tools (for example information systems, telemedicine, e-health)
- Working as a private practitioner and entrepreneurship

19. How well does your basic medical education correspond to your work?

- Very little
- Quite little
- To a moderate extent
- Quite well
- Very well

20. How satisfied are you with the teaching in hospital work you received during your basic medical education?

- Very dissatisfied
- Fairly dissatisfied
- Hard to say
- Fairly satisfied
- Very satisfied

21. How satisfied are you with the health centre teaching you received during your basic medical education?

- Very dissatisfied
- Fairly dissatisfied
- Hard to say
- Fairly satisfied
- Very satisfied

22. How well does the teaching you received during your basic medical education correspond to the work of a physician as regards the following topics?

1 = Very poorly, 2 = Rather poorly, 3 = To a moderate extent, 4 = Rather well, 5 = Very well

- Working as a hospital physician
- Working as a health centre physician
- Working on an inpatient ward
- Working at a clinic in specialist healthcare
- Working at a clinic in primary care
- Working at maternity and child welfare clinics
- School health care
- Occupational health care
- Rehabilitation
- Caring for the elderly
- Home nursing
- End-of-life care
- Pain management
- Dealing with addiction
- Environmental health care
- Administrative work
- Working as a private practitioner

**Specialist training**

23. What is your specialisation status?

- I have not decided whether to specialise or not
- I have decided not to specialise
- I have decided to specialise but am not yet sure in which specialty
- I have decided to specialise and chosen my specialty
- I am currently a specialist trainee
- I have completed my specialisation

If you are a specialist trainee or have completed your specialisation, please answer the following questions concerning your specialist education. Otherwise please jump to question 36.

24. In which specialty are you specialising or have already specialised?

Choose one specialty only. If you have several specialties, please choose **your latest specialty**.

Emergency Medicine

Anaesthesiology and Intensive Care Medicine

Endocrinology

Phoniatrics

Physical and Rehabilitation Medicine

Gastroenterology

Gastroenterological Surgery

Geriatrics

Dermatology and Allergology

Infectious Diseases

Cardiology

Respiratory Medicine and Allergology

Clinical Pharmacology and Pharmacotherapy

Clinical Physiology and Nuclear Medicine

Clinical Haematology

Clinical Chemistry

Clinical Microbiology

Clinical Neurophysiology

Otorhinolaryngology

Hand Surgery

Paediatric Surgery

Child Neurology

Child Psychiatry

Paediatrics

Sports Medicine

Obstetrics and Gynaecology

Nephrology

Neurosurgery

Neurology

Adolescent Psychiatry

Forensic Medicine

Forensic Psychiatry

Orthopaedics and Traumatology

Pathology

Clinical Genetics

Plastic surgery

Psychiatry

Radiology

Rheumatology

Ophthalmology

Internal Medicine

Oral and Maxillofacial Surgery

Cardiothoracic Surgery

Oncology

Public Health

Occupational Health

Urology

Vascular surgery

General Surgery

General Practice

Please answer all the following questions concerning your specialist education on the basis of **your latest specialty**.

25. In which year did you complete your specialist education? _________

26. If you are currently a specialist trainee:

- In which year were you accepted to study your current specialty? ______________
- In which year (estimate) will you complete your specialist education? ____________
- Estimate the proportion of your specialist education that you have now completed.
- Less than 50%
- At least 50%

27. In which training unit did you specialise or are you currently specialising?

- University of Helsinki
- University of Kuopio / University of Eastern Finland
- University of Oulu
- University of Tampere
- University of Turku
- Abroad, please specify

28. To what extent will/did the following factors influence your choice of specialty?

1 = Not at all, 2 = A little, 3 = Somewhat, 4 = Quite a lot, 5 = Very much

- Good example set by colleagues in the specialty
- Positive work experience in the specialty during my studies
- High quality specialisation programme
- Wide field
- Good prospects of finding work
- Opportunity to gain a good income
- Well-respected profession
- Opportunities to work in the private sector
- Opportunities for career advancement
- Opportunity to carry out research
- Opportunity to control the amount of work I do
- Good opportunity to balance family and work
- Small amount of on-call duty
- My personal skills and personality
- My chances of getting accepted for specialist education
- Geographical location of the place where specialist education is provided
- Chance

29. If you were starting your specialist education now, would you still choose the same specialty?

- No
- Yes

30. How satisfied are you with your specialist education as a whole?

- Very dissatisfied
- Fairly dissatisfied
- Hard to say
- Fairly satisfied
- Very satisfied

31. How well does your specialist education correspond to your work?

- Very little
- Quite little
- To a moderate extent
- Quite well
- Very well

32. How much instruction and guidance on the following topics did you receive during your specialist education?

1 = Far too little, 2 = Too little, 3 = The right amount, 4 = Too much, 5 = Far too much

Medical knowledge

- Diagnosis and treatment of diseases
- Examination and treatment procedures
- Patient safety

Learning new things

- Information search skills
- Critical evaluation of information
- Research
- Teaching

Interaction skills

- Meeting the patient
- Patient-centredness (patient’s needs and overall situation)
- Interacting with the patient’s relatives
- Written communication (patient records)

Collaboration skills

- Working in a group
- Group leadership
- Presentation skills
- Consultation skills
- Multidisciplinary collaboration

Professional attitude

- The profession’s values and commitment to those values
- Recognising the limits of my skills and acting accordingly
- Ethical considerations

Executive skills

- Effective use of time and prioritising different activities
- Health economics (appropriate use of resources)
- Personal professional advancement

Health promotion

- Health guidance and supporting patients in self-management
- Patient guidance methods
- Health promotion among the population

33. How much instruction and guidance on the following topics did you receive during your specialist education?

1 = Far too little, 2 = Too little, 3 = The right amount, 4 = Too much, 5 = Far too much

- Collaboration between different specialties
- Collaboration between primary care and specialist care
- Collaboration with social services
- Collaboration with different service sectors in the municipality (other than social services)
- Multiculturalism
- Physicians’ electronic tools (for example information systems, telemedicine, e-health)
- Working as a private practitioner and entrepreneurship

34. Estimate **how well** the following areas **were covered** during your specialist education:

1 = Very poorly, 2 = Rather poorly, 3 = To a moderate extent, 4 = Rather well, 5 = Very well

- Learning the diagnostic skills of my specialty
- Learning the diagnostic and treatment procedures of my specialty
- Guidance/support in clinical work (clinical instruction, consultation support)
- Guidance/support in professional growth and advancement
- The time allocated to instructors to provide instruction
- Systematic nature of education (defining learning goals, rotation between different locations)
- Monitoring and recording how well learning goals are achieved (e.g. log book, portfolio)
- Assessment of my skills and subsequent feedback to guide my learning
- Opportunity to prepare for the specialists’ examination
- Opportunity to carry out research
- Opportunity to perform administrative work and development
- Opportunity to study administration/management related to my specialisation
- In-service training
- Opportunity to obtain training outside my place of work
- Theoretical courses organised by the university
- Theoretical courses organised by other parties

35. The assessment and feedback you received during your specialist education is/was...

Answer by choosing one of the following options: 1 = completely disagree, 2 = partly disagree, 3 = hard to say, 4 = partly agree, 5 =completely agree

- systematic and regular 1 2 3 4 5
- based on set goals 1 2 3 4 5
- constructive 1 2 3 4 5
- adequate 1 2 3 4 5

36. One specialist in five has more than one specialty.

- Are you a licensed specialist in more than one specialty?

1 = No ⇒ Go to question 37.

- 2 = Yes ⇒ If yes, is one of your specialties General Practice?

1 = No, 2 = Yes

**Doctoral thesis and other research**

37. What is your situation as regards a doctoral thesis?

- I have not decided whether to study for a doctorate or not
- I have decided not to study for a doctorate
- I plan to start a doctoral thesis but have not yet decided on the topic
- I plan to start a doctoral thesis and have chosen my topic
- I am working on my doctoral thesis
- I have passed my doctoral viva

38. Are you conducting scientific research without aiming for a doctorate?

- Yes
- No

If you are not conducting research, please jump to question 39.

39. How do you conduct research? You can choose several options.

- I conduct research full-time
- I conduct research part-time
- I conduct research outside my day job (for example during evenings and weekends)
- I participate in research projects (for example clinical studies)
- I supervise research
- I have authored a scientific article during the past three years
- In other ways, please specify ___________

**Management**

40. Is your immediate superior?

- A physician
- Other
- I have no superior

41. To what extent are you interested in working in health care management?

- Not at all
- A little interested
- Quite interested
- Very interested

42. Do you act as a superior to others?

- Yes
- No

43. How much management training have you had? You can choose several options.

- None
- 20 hours during my specialist education (included in specialist education prior to 2009)
- 10 credits during my specialist education
- 30 credits during my specialist education
- Diploma in Health Administration (prior to 2003)
- Finnish Medical Association’s leadership education for specialists (ELJ) (30 credits)
- A 60 credit syllabus in Health and Social Management (PD)
- MBA or similar (80 to 100 credits)
- Other, please specify Scope ___________________________________

**Licence to work as a physician**

44. Should physicians’ licences be fixed-term (so-called recertification)?

- No
- Yes
- Don’t know

45. If physicians’ licences were fixed-term, should any of the following be considered as prerequisites for the licence? 1 = No, 2 = Yes

- Sufficient clinical work
- Participation in complementary medical education
- Written or oral examination
- Demonstration of skills / objectively structured clinical examination (OSCE)
- Documentation of learning at work
- Peer review by colleagues
- Other, please specify ______________

**Respondent’s background information**

46. Gender

- Male
- Female
- Other
- I prefer not to say

47. Year of birth ____________

48. Marital status

- Unmarried
- Cohabiting
- Married or in a registered same-sex partnership
- Divorced, separated
- Widowed
- I prefer not to say

49. What is your spouse’s/partner’s education?

- Physician
- Other health care education
- Other education

50. Number of children ____________

51. Your mother’s education

- Physician
- Other health care education
- Other education, please specify ______________

52. Your father’s education

- Physician
- Other health care education
- Other education, please specify ______________
